# Vascular and lung function related to ultrafine and fine particles exposure assessed by personal and indoor monitoring: a cross-sectional study

**DOI:** 10.1186/1476-069X-13-112

**Published:** 2014-12-15

**Authors:** Yulia Olsen, Dorina Gabriela Karottki, Ditte Marie Jensen, Gabriel Bekö, Birthe Uldahl Kjeldsen, Geo Clausen, Lars-Georg Hersoug, Gitte Juel Holst, Aneta Wierzbicka, Torben Sigsgaard, Allan Linneberg, Peter Møller, Steffen Loft

**Affiliations:** Section of Environmental Health, Department of Public Health, Faculty of Health and Medical Sciences, University of Copenhagen, Øster Farimagsgade 5A, 1014 Copenhagen, Denmark; International Centre for Indoor Environment and Energy, Department of Civil Engineering, Technical University of Denmark, Nils Koppels Alle 402, 2800 Lyngby, Denmark; Division of Ergonomics and Aerosol Technology, Lund University, P.O. Box 118, SE-221 00 Lund, Sweden; Department of Public Health, Section of Environment, Occupation & Health, University of Aarhus, Bartholins Allé 2, 8000 Aarhus, Denmark; Research Centre for Prevention and Health, Capital Region of Denmark, Glostrup University Hospital, Nordre Ringvej 57, 2600 Glostrup, Denmark; Department of Clinical Experimental Research, Glostrup University Hospital, Nordre Ringvej 57, 2600 Glostrup, Denmark; Department of Clinical Medicine, Faculty of Health and Medical Sciences, University of Copenhagen, Blegdamsvej 3B, 2200 Copenhagen, Denmark

**Keywords:** Indoor air pollution, Ambient air pollution, Ultrafine particles, Personal monitoring, Microvascular function, Inflammation, Lung function

## Abstract

**Background:**

Exposure to ambient air particulate matter (PM) has been linked to decline in pulmonary function and cardiovascular events possibly through inflammation. Little is known about individual exposure to ultrafine particles (UFP) inside and outside modern homes and associated health-related effects.

**Methods:**

Associations between vascular and lung function, inflammation markers and exposure in terms of particle number concentration (PNC; d = 10-300 nm) were studied in a cross-sectional design with personal and home indoor monitoring in the Western Copenhagen Area, Denmark. During 48-h, PNC and PM_2.5_ were monitored in living rooms of 60 homes with 81 non-smoking subjects (30-75 years old), 59 of whom carried personal monitors both when at home and away from home. We measured lung function in terms of the FEV_1_/FVC ratio, microvascular function (MVF) and pulse amplitude by digital artery tonometry, blood pressure and biomarkers of inflammation including C-reactive protein, and leukocyte counts with subdivision in neutrophils, eosinophils, monocytes, and lymphocytes in blood.

**Results:**

PNC from personal and stationary home monitoring showed weak correlation (r = 0.15, p = 0.24). Personal UFP exposure away from home was significantly inversely associated with MVF (1.3% decline per interquartile range, 95% confidence interval: 0.1-2.5%) and pulse amplitude and positively associated with leukocyte and neutrophil counts. The leukocyte and neutrophil counts were also positively and pulse amplitude negatively associated with total personal PNC. Indoor PNC and PM_2.5_ showed positive association with blood pressure and inverse association with eosinophil counts.

**Conclusions:**

The inverse association between personal exposure away from home and MVF is consistent with adverse health effects of UFP from sources outside the home and might be related to increased inflammation indicated by leukocyte counts, whereas UFP from sources in the home could have less effect.

## Background

Deleterious effects of ambient air pollution and especially particulate matter (PM) are documented by a large and continuously growing number of studies [1–6]. PM consists of a mixture of solid and liquid particles of organic and inorganic origin suspended in the air, largely varying in size, chemical composition, and toxicity due to continuously undergoing multiple physical and chemical processes [7]. Whereas the mass concentrations of PM with diameter below 10 μm (PM_10_) and 2.5 μm (PM_2.5_) are under regulation, there are currently no guideline values for ultrafine particles (UFP) with diameter below 100 nm, mostly formed during combustion processes, such as in diesel engines or burning candles [7, 8]. The UFP mass is small compared to the larger particulate fractions and UFP exposure is poorly represented by mass concentration, leaving particle number concentration (PNC) and surface area as potentially more accurate defining metrics emphasizing their hazardous nature [1, 9]. The vast body of epidemiological evidence on health effects of PM relies on exposure assessment based on monitored or modeled ambient levels of mainly PM_10_ and PM_2.5_ [1]. The UFP levels in the studies that found association with health outcomes show high spatial and temporal variation demonstrating the necessity of advanced exposure assessment models [1].

Outdoor particles can infiltrate to indoor air through open doors and windows, building ventilation system, gaps and cracks in the building envelope [10–12]. In addition, UFP and fine particles can also be released from human activities occurring indoors. Combustion processes, such as candle burning, cooking, smoking, working space heaters, woodstoves, fireplaces, and gas stoves as well as cleaning, use of house care products, and working office equipment can substantially raise and further dominate indoor particle levels [13–16]. Therefore, integrated human exposure to UFP and fine particles is highly complex as it encompasses exposure to particles from both outdoor and indoor sources [8, 17–19]. Considering that people spend most of their time indoors, knowledge on the relevance in terms of health effects for the particle exposure occurring at home is important for targeted abatement strategies.

Observations of short-term effects on vascular and lung function as well as biomarkers of inflammation or oxidative stress support epidemiological findings by delineating mechanisms of actions of PM mediated health effects [2, 20–23]. Whereas a large number of studies show negative association between ambient exposure to traffic-related pollution and lung function in children and in adults, few studies found an association between indoor PM and lung function [1, 24]. Vasomotor dysfunction, a predictor of cardiovascular events, has been associated with exposure to ambient air PM and to high concentrations of diesel exhaust particles in healthy subjects, elderly, and susceptible groups [21–23, 25, 26]. Microvascular function (MVF) can be non-invasively assessed as an index of vasomotor function by means of Peripheral Arterial Tonometry (PAT), which is applicable in nonclinical settings such as private houses or offices and has been shown predictive of cardiovascular events in one study [27]. Short-term chamber exposure studies with duration from 3-h to 24-h have not found a significant association between exposure to ambient air or wood smoke and MVF in young subjects, whereas 2 to 14 days with air filtration in homes decreased PM_2.5_ levels and improved MVF in middle-aged and elderly subjects [28–30]. The PAT measurement also provides baseline pulse amplitude that recently has been associated with air pollution [31].

Systemic inflammation, which is regarded as an intermediate step from the respiratory particle exposure to cardiovascular effects, has been assessed by biomarkers including C-reactive protein (CRP) and counts of leukocytes, especially neutrophils and other granulocytes [2, 20]. Long- and short-term exposure studies, particularly with children and healthy adults, show positive associations between ambient PM and CRP, while there is less consistency in associations with leukocytes and very limited data for indoor air [32]. Indoor biomass smoke with high PM concentrations was associated with increased CRP levels in a cross-sectional study of Indian women [33]. One intervention study associated decrease in indoor particle exposure with decrease in CRP levels, whereas similar studies found no effect on CRP [28, 30, 34].

Accurate individual exposure assessment in- and outside the home requires time-resolved personal monitoring. Small portable instruments can measure PNC and personal exposure appears more strongly correlated to indoor than ambient PNC levels [8, 17, 35]. However, the particles’ characteristics differ substantially in relation to the sources, which can influence their potential toxicity. Indeed, a personal monitoring study found ambient PNC, mainly traffic derived, to be three times more potent in oxidative stress-induced DNA damage in mononuclear leukocytes than indoor PNC [36]. However, there is a lack of knowledge on the difference in hazard between the PNC exposure in home and out of home with respect to vascular and lung function, as well as to inflammatory responses.

The aim of the present study was to associate PNC continuously monitored through 48-h using a stationary monitor in the home and a personal monitor worn both in the home and away from home, with MVF, pulse amplitude, blood pressure, lung function and biomarkers of inflammation among 60 healthy subjects and 23 spouses. The hypothesis was that the exposure away from home would be more strongly associated with the health-related outcomes than the exposure in the home, assessed by portable and stationary instruments.

## Methods

### Study population

The study population was recruited from follow up of The Health2006 cohort [37]. Participants of the Health2006 cohort consist of a random sample living in the South-Western part of the greater Copenhagen area (a total of 3471 persons with Danish citizenship and born in Denmark) aged 18-69 years in 2006. The cohort was set up for population-based research of prevalence and risk factors of chronic diseases (coronary heart disease, diabetes, musculoskeletal disorders, chemical intolerance, osteoporosis, asthma, allergy, chronic lung diseases and mental disorders).

Inclusion criteria for the present study were no history of smoking and residence at the same non-smoking address for not less than the last six months. People taking lipid lowering or vasoactive medication were excluded. About 300 letters were sent by post to the eligible participants of the cohort. Their healthy spouses were also invited to participate. A total of 83 persons from 60 addresses (20% response) participated in the study. Two of the subjects were excluded from the analyses of health-related outcomes due to use of angiotensin II receptor antagonist revealed after sample collection and recent infection treated with antibiotics and resulting in a high CRP level. The characteristics of the 81 participants with health outcomes are presented in Table 1.

**Table 1:** **Characteristics of the study participants (numbers or mean ± SD)**

	Men	Women	Total
Number	41	40	81
Age (years)	54 ± 12	49 ± 11	51 ± 12
Height (cm)	179 ± 7	167 ± 7	173 ± 9
Weight (kg)	79 ± 10	64 ± 10	72 ± 12
Body mass index (kg/m^2^)	25 ± 3	23 ± 3	24 ± 3
Hemoglobin (mmol/L)	10.8 ± 1.5	9.9 ± 1.5	10.4 ± 1.5
Hb_A1c_ (hemoglobin A1c; (mmol/mol)	37 ± 10.3	33.6 ± 4.0	35.3 ± 8.0
Glucose (mmol/L)	5.1 ± 11	4.7 ± 1.2	4.9 ± 1.2
Total cholesterol (mmol/L)	4.2 ± 0.9	4.1 ± 0.8	4.1 ± 0.8
LDL cholesterol (mmol/L)	2.6 ± 0.7	2.3 ± 0.7	2.4 ± 0.7
HDL cholesterol (mmol/L)	1.2 ± 0.3	1.6 ± 0.4	1.4 ± 0.4
Triglycerides (mmol/L)	1.0 ± 0.5	0.7 ± 0.3	0.9 ± 0.4
Personal monitoring (number of participants)	36	23	59
Time spent away from home by personal monitoring participants during the experiment (%)	29 ± 14	29 ± 14	29 ± 14

A written informed consent was obtained from each participant. The study was reviewed and approved by The Committees on Health Research Ethics in the Capital Region of Denmark (file no. H-4-2012-173).

### Exposure assessment

The study started on the 20^th^ of February 2013 and was finished on the 31^st^ of May 2013. Exposure monitoring for each home and person was performed during about 2 days. Seven Aerasense NanoTracer particle counters (Philips) were used for both measurements (home microenvironment and personal monitoring) of PNC. According to the producer’s description the instrument measures PNC for particles in 10 nm to 300 nm range with 16 sec. time resolution.

All seven newly calibrated NanoTracers used in the experiment were extensively tested against each other and against a Scanning Mobility Particle Sizer (SMPS 3934, TSI Inc., USA) in an aerosol chamber. The measurements used for the comparison were performed over several days during which cooking, candle, and incense burning events took place. These chamber measurements also included periods of low concentrations without sources present, in order to represent concentrations occurring for example in homes during unoccupied periods. The average NanoTracer-to-SMPS concentration ratio varied between 0.69 and 1.22 for the seven instruments (average of all instruments was 0.93) and this ratio was used as a correction factor for each instrument in order to compensate for inter-equipment variation. The correction factors were applied to the particle concentrations obtained from the corresponding instruments, in order to obtain the final results. A similar set of validation experiments with our NanoTracer instruments have been described in detail elsewhere [13], whereas further accuracy information is available from other research groups [35]. The PNC obtained from the instruments are considered a reasonably proxy of UFP because particles larger than 100 nm generally contribute very little.

One NanoTracer along with a PM_2.5_ sampler (see below) was placed in the living room. Average indoor PNC from the monitoring in the homes was calculated based on an average of 47-h data per home. An identical NanoTracer was carried in a backpack by 59 participants (one person from each home) for personal monitoring throughout the time of the experiment including while being at home. The NanoTracer for personal monitoring was connected to a sampling tube that was led outside of the backpack. To optimize personal exposure monitoring, the participants were asked to keep the backpack at their bedside, while they were sleeping. On a few occasions the personal monitor ran out of power before the end of the measurement. The average PNC during the entire personal monitoring period (total personal PNC) was calculated from the available data, which corresponds to ≈ 43-h on average. Further, the integrated personal exposure was calculated as PNC multiplied by time for the periods when the person was at home (personal exposure at home) and away from home (personal exposure away from home) according to the information on occupancy times at home from the individual diaries.

The indoor PM_2.5_ mass concentrations were measured gravimetrically on Fluoropore Membrane PTFE filters (37 mm; pore size, 1.0 μm; Millipore, Billerica, MA, USA). The setup consisted of a cyclone sampling head GK 2.05-KTL (BGI Inc, Waltham, MA, USA) with a cut off diameter of 2.5 μm, a filter and a sampling pump. The airflow through the sampling filter was adjusted to 4 L/min at the start of each measurement session and it was checked again at the end of the measurement period. Before and after sampling the filters were kept at constant temperature (22°C) and relative humidity (50%) for 24-h before being weighed. The average airflow was used to calculate the average PM_2.5_ concentration in each residence during the measurement period. In 8 homes the PM_2.5_ sampling was incomplete either due to failure of the pump or because the pump was intentionally stopped by the occupants disturbed by the noise.

### Assessment of health-related outcomes

On the third day of the experiment, after approximately 48-h of exposure monitoring and at around the same time of day when the instruments were being uninstalled, the physiological function tests were performed and material for biomarker analysis was collected.

Lung function: Spirometry test was performed using the NDD Easy One Plus spirometer (ndd Medical Technologies, Zurich Switzerland) in accordance with the European Respiratory Society (ERS) standards and American Thoracic Society (ATS) standards summarized by Miller et al. [38]. The largest from at least three acceptable manoeuvres of forced expiratory volume in the first second (FEV_1_) and forced vital capacity (FVC) were recorded and used for calculation of the FEV_1_/FVC ratio.

Microvascular function (MVF): Prior to each MVF measurement, resting blood pressure was measured by means of a Welch Allyn DuraShock DS54 manometer (Welch Allyn GmbH & Co. KG, Deutschland). The cuff was placed above the elbow on one arm, which was afterwards used for the occlusion in the MVF measurement. MVF was measured by means of specially designed finger probes (a pulse amplitude tonometry, EndoPAT device) placed on the tip of index fingers of the subject (EndoPAT2000; Itamar Medical Ltd, Cesaria, Israel) as described previously [28]. Baseline pulse amplitude measurements were performed for 5 minutes, after which the blood pressure cuff was inflated in order to stop blood flow through the brachial artery, inducing ischemia in the test arm. Occlusion of pulsatile arterial flow was confirmed by the reduction of the PAT tracing to zero. After 5 minutes of occlusion, the cuff was deflated, followed by reactive hyperemia, and PAT measurements continued to be recorded for further 5 minutes. The MVF score was calculated by a computer algorithm automatically normalized for the baseline PAT signal and for the signals of the contralateral arm that served as a control.

Biomarkers in blood: On the day of the home visit, peripheral venous blood samples were collected in EDTA tubes. Within 4 hours after the draw, blood was analyzed for hemoglobin, leukocytes and their subtypes, and separated plasma was stored at -80°C for later CRP analysis at the Department of Clinical Biochemistry, Copenhagen University Hospital. Measurements of hemoglobin, leukocytes lymphocytes, and monocytes counts, were performed by an automatic portable hematological analyzer Chempaq XBC (Chempaq A/S, Denmark), calibrated automatically before each measurement. Neutrophils, eosinophil and basophil counts were measured by the HemoCue leukocyte system (HemoCue AB, Sweden).

### Statistical analysis

In order to assess the associations between individual exposure and MVF, lung function, and inflammatory markers, i.e. leukocytes, and CRP, multiple linear regression analysis was applied using the STATA 13 program package (StataCorp LP, College Station, TX, USA). Regression models were based on average personal PNC during the whole measurement period or on the integrated exposure, determined separately for the periods when the subjects were in the home and away. Since the subjects living at the same address share the measure of exposure, which may violate the principle of predictor variable independence between subjects, a generalized estimating equations (GEE) model was used for PNC and PM_2.5_ measured by the stationary indoor monitors. For outcomes with more than one exposure metric as significant predictor two-pollutant models were run. All models were adjusted for possible confounders, i.e. sex, age, and BMI, the latter two as continuous variables.

## Results

A summary of the exposure data is presented in Table 2. The subjects who carried a NanoTracer spent on average 29% of the time away from home. The personal average PNC over the total time of the experiment was more strongly correlated with the average personal PNC when away from home (r = 0.92) than with the average personal PNC when at home (r = 0.37). The average personal PNC when inside the home was significantly but modestly correlated with PNC (r = 0.41) and PM_2.5_ (r = 0.44) levels measured by stationary monitors in the home during the whole period. The personal exposure at home and away from home based on PNC and time spent at home and away followed the same trends as for the corresponding personal PNC.

**Table 2:** **Correlations between exposure variables**

		Personal monitoring - PNC			Personal exposure		Stationary indoor (monitoring (home)	
		Total period (10^3^/cm^3^)	While at home (10^3^/cm^3^)	While away from home (10^3^/cm^3^)	At home (10^3^/cm^3^)*h	Away from home (10^3^/cm^3^)*h	PNC (10^3^/cm^3^)	PM_2.5_(μg/m^3^)
**N**		59	59	59	59	59	81	72
**Median** **(5** ^**th**^ **, 95** ^**th**^ **percentile)**		9.2	8.3	8.8	285.9	125.9	8.4	12.2
		(4.5, 36.5)	(3.3, 36.7)	(2.8, 55.5)	(93.2, 1.041)	(14.3, 927.8)	(3.3, 23.0)	(7.5, 24.1)
**Personal monitoring - PNC**	**Total**	1.000						
	**While at home**	0.45*	1.000					
		(0.00)						
	**While away from home**	0.93*	0.10	1.000				
		(0.00)	(0.43)					
**Personal exposure**	**At home**	0.37*	0.97*	0.02	1.000			
		(0.00)	(0.00)	(0.85)				
	**Away from home**	0.92*	0.09	0.99*	−0.001	1.000		
		(0.00)	(0.50)	(0.00)	(0.99)			
**Stationary indoor monitoring**	**PNC**	0.15	0.41*	0.01	0.36*	0.02	1.000	
		(0.24)	(0.00)	(0.94)	(0.00)	(0.86)		
	**PM**_**2.5**_	0.22	0.44*	0.03	0.38*	0.05	0.49*	1.000
		(0.12)	(0.00)	(0.84)	(0.00)	(0.70)	(0.00)	

The variables include personal PNC (during total measurement period, while at home and while away from home), personal exposure occurring at home and away from home, and stationary PNC and PM_2.5_ measured in the home.

Values are medians (5^th^, 95^th^ percentiles) and Pearson product moment coefficients (p-values). * p < 0.05.

PNC: particle number concentration.

Descriptive statistics for the health-related outcome variables are presented in Table 3. The associations between the health-related outcomes and the exposure levels estimated as percent change per inter-quartile range (IQR) are presented in Table 4. The associations were based on 57 subjects with personal monitoring data and 81 with home monitoring data. Personal monitoring data for two subjects were excluded from the analyses because of vasoactive drug use and recent infection. PM_2.5_ data were available for 72 subjects.

**Table 3:** **Microvascular function (MVF), pulse amplitude, blood pressure, lung function (FEV**_**1**_ **/FVC), and biomarkers of inflammation among 81 study participants**

Biomarkers	Total (n = 81)	Men (n = 41)	Women (n = 40)
MVF	1.9 (1.1, 3.1)	1.9 (1.1, 3.1)	1.9 (1.2, 3.4)
Log pulse amplitude	6.1 (4.3, 7.0)	6.4 (5.0, 7.0)	5.8 (4.2, 6.8)
Systolic blood pressure (mmHg)	120 (90, 150)	130 (110, 150)	120 (90, 140)
Diastolic blood pressure (mmHg)	70 (60, 90)	70 (60, 100)	70 (50, 85)
C-reactive protein (mg/L)	0.5 (0.1, 7.4)	0.5 (0.3, 7.5)	0.5 (0.1, 6.2)
Leukocytes (x10^9^cells/L)	5.6 (3.3, 8.5)	5.2 (3.3, 8.5)	5.6 (3.5, 8.1)
Lymphocytes (x10^9^cells/L)	1.7 (1.1, 2.6)	1.6 (1.0, 2.5)	1.7 (1.1, 3.2)
Monocytes (x10^9^cells/L)	0.5 (0.2, 0.8)	0.5 (0.2, 0.7)	0.5 (0.2, 0.9)
Neutrophils (x10^9^cells/L)	2.9 (1.6, 4.8)	2.8 (1.6, 5.6)	3.0 (1.7, 4.6)
Eosinophils (x10^9^cells/L)	0.1 (0.0, 0.4)	0.1 (0.0, 0.4)	0.1 (0.0, 0.3)
FEV_1_/FVC	0.80 (0.7, 0.9)	0.80 (0.7, 0.9)	0.80 (0.7, 0.9)

Values are medians (5^th^, 95^th^ percentiles). MVF, Microvascular function; FEV_1_, Forced expiratory volume 1s (L); FVC, Forced vital capacity (L).

**Table 4:** **Associations between exposure and the health-related outcomes**

	Personal monitoring			Stationary indoor monitoring (home)	
	PNC total period (10^3^/cm^3^)	Exposure at home (10^3^/cm^3^)*h	Exposure away from home (10^3^/cm^3^)*h	PNC (10^3^/cm^3^)	PM_2.5__(μg/m^3^)
Number of subjects	N = 57	N = 57	N = 57	N = 81	N = 72
IQR	6.9	325.3	141.5	5.1	7.4
MVF	−2.6 (-5.2, 0.1)	2.4 (-5.2, 10.5)	−1.3* (-2.5, -0.1)	−0.03 (-2.4, 2.4)	6.7 (-0.4, 14.3)
Baseline pulse amplitude	−9.6* (-15.7, -3.0)	−15.11 (-30.4, 3.6)	−3.4* (-6.4, -0.3)	−1.1 (-8.5, 7.0)	−13.6 (-31.3, 8.6)
Systolic blood pressure	0.7 (-0.6, 2.0)	1.3 (-2.1, 4.8)	0.3 (-0.3, 0.8)	2.4* (0.9, 3.8)	4.6 (-0.003, 9.2)
Diastolic blood pressure	0.3 (-0.5, 1.2)	1.3 (-1.1, 3.7)	0.1 (-0.3, 0.5)	2.3* (1.4, 3.2)	2.8 (-0.4, 6.0)
C-reactive protein	−1.2 (-11.7, 10.7)	−3.1 (-28.5, 31.3)	−0.5 (-5.3, 4.6)	6.1 (-2.2, 15.0)	−4.8 (-14.8, 6.4)
Leukocytes	3.1* (0.3, 5.9)	−0.7 (-7.9, 7.1)	1.5* (0.3, 2.7)	1.1 (-1.2, 3.5)	1.6 (-5.8, 9.5)
Lymphocytes	1.7 (-1.4, 5.0)	2.8 (-5.6, 11.9)	0.6 (-0.7, 2.1)	2.4 (-1.1, 6.0)	13.2* (1.3, 26.6)
Monocytes	−0.8 (-4.6, 3.1)	−6.4 (-15.6, 3.9)	−0.03 (-1.7, 1.7)	1.4 (-3.5, 6.6)	5.8 (-7.6, 21.0)
Neutrophils	5.5* (1.7, 9.4)	1.9 (-8.3, 13.4)	2.4* (0.8, 4.1)	0.7 (-2.6, 4.1)	−3.7 (-13.1, 6.6)
Eosinophils	−3.3 (-8.6, 2.4)	−9.0 (-23.6, 8.6)	−1.0 (-3.4, 1.5)	−5.2* (-9.8, -0.4)	−16.3* (-27.1, -3.9)
FEV_1_/FVC	−0.6 (-1.4, 0.2)	−1.0 (-3.1, 1.2)	−0.2 (-0.6, 1.1)	0.5 (-0.1, 1.1)	0.7 (-1.2, 2.6)

Percent change (95% confidence interval) in outcome levels associated with an interquartile range (IQR) increase in personal particle number concentration (PNC) monitored for the whole period and personal exposure (concentration times time) at home and away from home estimated with regression models and with stationary home indoor PNC and PM_2.5_ estimated with generalized estimation equations on the natural logarithm of the outcomes with adjustment for age, gender, and BMI, respectively.

MVF, Microvascular function; FEV_1_, Forced expiratory volume 1s (L); FVC, Forced vital capacity (L). * p < 0.05.

MVF was significantly inversely associated with the personal exposure away from home. Similar associations were observed for the total personal PNC, but these results were not significant. Pulse amplitude also showed significant inverse association with personal exposure away from home as well the total personal PNC. Significant positive associations were found between total leukocyte and neutrophil counts and both total personal PNC and personal exposure away from home. There was no such association for the personal exposure at home. In two-pollutant models including personal exposure at home and away from home, all these significant associations with personal exposure were retained (data not shown). Lung function showed some inverse association with personal monitoring variables, although these were not statistically significant. CRP and monocyte counts did not show significant associations with any exposure metric.

Stationary indoor PNC and PM_2.5_ in the homes showed statistically significant inverse association with eosinophil counts. In a two-pollutant model only the association with PM_2.5_ remained significant (data not shown). A similar, but not significant, relationship was observed between eosinophil counts and the personal monitoring variables. Lymphocyte counts were positively associated with all exposure variables, but the association was significant only with respect to PM_2.5_ concentrations in the homes. The stationary indoor PNC also showed significant positive association with systolic and diastolic blood pressure. These significant associations with PNC were retained in a two-pollutant model including indoor PM_2.5_, which in single-pollutant models showed borderline significant associations with blood pressure.

## Discussion

The main findings of this cross-sectional study were that personal exposure occurring away from home showed inverse association with MVF as well as pulse amplitude and positive association with leukocyte and neutrophil counts. Personal exposure at home and stationary PNC inside the home did not show such associations, although blood pressure was associated with stationary indoor PNC. The results are consistent with the well-known cardiovascular effects of traffic-generated UFP, which are mainly encountered outside. The results suggest that particles from sources in the home might be less harmful.

There was no trend of inverse associations between MVF and indoor stationary PNC or PM_2.5_ in our study, although blood pressure did show association with stationary indoor PNC. Two indoor intervention studies with particle filtration over 2-7 days indicated improvement in MVF measured with the EndoPat, but no effect on blood pressure [29, 30]. Although one of the studies measured PNC levels in the home, it was reported that the particulate volume, which is closely related to mass concentration, was a stronger predictor of the improved MVF than PNC. In the other study, exposure was measured only in terms of indoor PM_2.5_ mass concentration. Similarly, we have observed earlier that particle filtration in the home for 2 weeks was only associated with improvement in MVF in relation to the actual decrease in PM_2.5_ in the bedroom [28]. These intervention studies provided sufficient contrast in exposure to PM_2.5_ mass concentration and subjects were staying at home most of the time whereas the cross-over design limited the influence from potential inter-individual confounders. A fourth intervention study with one week indoor air filtration showed no effect on MVF, which might be due to the young age of the study population, tobacco smoke being the main source of PM in the homes, and/or the use of electrostatic filters rather than the high efficiency particulate adsorption filters, that were used in the other intervention studies [39]. In addition, a number of studies with young individuals found no effect of short-term exposure to traffic and wood combustion particles on MVF measured by EndoPat within a few hours after exposure [34, 40, 41]. One study found an inverse association between MVF and ambient PM_2.5_ levels during the two preceding days [40], whereas more recent studies found no such associations [31, 42]. Possibly, longer or more intense exposure is required to detect the effects.

Although some of the personal exposure away from home also occurred in indoor setting, the significant inverse association with MVF may support the notion of deleterious effect of traffic- and diesel engine-generated particles on endothelial function found in a number of studies with controlled exposure at high levels [21, 22]. This is further supported by our recent finding of inverse association between 48-h PNC in ambient air and MVF in a cross-sectional study of citizens from central Copenhagen [42], although another study with a much wider catchment area in USA found no such association [31]. The present finding of a parallel inverse association between personal exposure away from home and pulse amplitude suggests that vasoconstriction might play a role, although the only study reporting this outcome in relation to ambient air pollution found positive association [31]. The highest dose intensity for PNC was encountered for men during commuting in a personal exposure study assessing time-activity patterns [35]. However, it should be borne in mind that the period, indicated as “away from home”, does not solely represent the time spent outdoors, but also includes periods spent in other indoor environments. Our findings indicate that there can be adverse effects on MVF of exposure to relatively low number concentrations of UFP encountered in daily life away from home (median of the 59 subjects’ average PNC: 8800 particles/cm^3^ of which >95% are UFP). This might be related to systemic oxidative stress which has been indicated by DNA base oxidation at similar UFP levels encountered during commuting in Copenhagen, Denmark [36]. Moreover, the results also emphasize the power of combining personal time-resolved exposure monitoring with a well-established measure of a key function in vascular disease employing non-invasive equipment applicable in field studies.

Pulmonary and systemic inflammations are considered to be important mechanisms in PM-induced cardiovascular disease [2]. We found signs of an inflammatory response in terms of increased total leukocyte and neutrophil counts associated with the personal exposure away from home, i.e. the same exposure metric associated with reduced MVF. No significant association between lung function and any exposure metric was observed. Short-term effects of traffic-related UFP exposure on lung function might be detectable mainly in susceptible adult subjects with, for instance, asthma [43]. We have very recently in another cross-sectional study performed in the winter found that lung function and markers of systemic inflammation were adversely associated with stationary PNC in the home dominated by candle burning, whereas the present study occurred in the spring with much less candle burning [42]. Indoor UFP exposure also appeared to be associated with inflammation and reduced lung function in children [24]. Some panel studies of risk groups showed associations between leukocyte counts and PNC and/or PM_2.5_ [1, 44, 45], whereas controlled exposures, including healthy subjects, did not find such associations [25, 26, 46–48]. The statistically significant inverse association between both PM_2.5_ and PNC monitored in the home and eosinophil counts in the blood could have occurred by chance but might also suggest either lower allergen exposure or stronger allergen tolerance associated with UFP exposure in the home [49], which has also been shown in relation to smoking [50].

Most studies on associations between short-term exposure to UFP or PM_2.5_ and CRP as a marker of inflammation were conducted using ambient PM concentrations measured at stationary monitoring stations, which is a poor proxy for personal exposure [32]. However, earlier studies with 24-h exposure to traffic derived UFP revealed no significant association with CRP, which is consistent with our findings [34, 51]. Some studies report 1- to 28- days lag for CRP levels increase, while some long-term studies revealed increase in CRP levels in highly exposed populations [32].

Although stationary monitoring of indoor and ambient particle concentrations can provide important information, personal monitoring represents the most accurate method to quantify individual exposure [8, 17, 35]. We observed a high correlation between total personal PNC during the whole measurement period and personal exposure away from home, despite the fact that 70% of the experiment time was spent in the home. It highlights the importance of exposure away from home, which can occur both outdoors and in various types of indoor environments, including vehicles. The weaker correlations between total personal PNC and personal exposure at home or the stationary indoor PNC in the living room, suggest that UFP in the home contributed relatively little to the total personal exposure in the present study. Indeed, it supports the use of time-resolved personal monitoring, although PNC measurements do not describe chemical composition of particles, nor do they identify sources of importance for health effects. For instance, separate indoor and outdoor personal exposure to UFP during 24-h periods in a panel study with 6 repeated measurements with 14 subjects suggested that the outdoor particles were around three times more potent in terms of increased levels of oxidative stress-induced damage to DNA in leukocytes, compared to indoor particles [36]. Some chemical composition characteristics such as the level of elemental carbon, polycyclic aromatic hydrocarbons or transition metals are typical for traffic generated particles and can be measured in PM_2.5_ sampled on filters over a period of time, but without time resolution. Indeed, the level of vanadium in personal PM_2.5_ filter samples were more closely related to oxidatively damaged DNA in leukocytes than was the total PM_2.5_ mass [52]. Individual differences in antioxidant defenses may also explain differences in susceptibility to PM exposure and contribute to heterogeneity in associations with outcomes [53].

The participants were people with no history of smoking, living in non-smoking homes. Hence, the presence of tobacco smoke, which is a major source of fine and ultrafine particles, was highly unlikely in the homes of the participants. Thus, cooking and candle burning may have been the main sources of UFP in the homes, although penetration of ambient particles from outdoors could contribute to indoor levels as well [8]. Indeed, in a recent study, candle burning, which is popular during wintertime in Denmark, was responsible on average for nearly 60% of the residential daily integrated UFP exposure [13].

Our study has a number of limitations. We had no information on the activities of the participants during the time they spent away from home. Such information could allow us to further assess associations between outcomes and specific exposures, such as those occurring in traffic or in other environments with potentially relevant sources. The cross-sectional design is susceptible to confounding from individual risk factors, including those related to socioeconomic status, which was not included in the analysis [54]. The associations between exposure and outcomes were relatively weak. We tested multiple associations between exposures and outcomes with inherent risk of chance findings. A larger sample size would have increased the statistical power and would allow adjustment for multiple testing and covariates. However, some of the outcomes in our data could be related to the same mechanism of action, which makes adjustment problematic. It is likely that the participation rate of 20% among the invited homes provided participants who were more concerned about their health. Although some systematic selection bias is possible, this is not likely to severely limit the generalization of the results.

## Conclusion

The negative association between personal UFP exposure away from home and MVF supports the hypothesis of negative effects of ambient particle air pollution on cardiovascular health, whereas indoor generated particles might have smaller effects. The adverse effects could be related to inflammation as suggested by a positive association between personal exposure to UFP occurring away from home and leukocyte and neutrophil counts. The results underline the benefit of using personal monitoring for elucidating health effects of particles and warrant future research with longer exposure monitoring, better source characterization by detailed time-activity patterns, chemical characterization, repeated outcome measurements and considering seasonal variation.

## Abbreviations

BMI: Body mass index
CRP: C-reactive protein
FEV1: Forced expiratory volume in 1 second
FVC: Forced vital capacity
MVF: Microvascular function
PM_2.5_: Particulate matter with aerodynamic diameter less than 2.5 μm
PNC: Particle number concentration
UFP: Ultrafine particles.

## References

[1] Ruckerl R, Schneider A, Breitner S, Cyrys J, Peters A (2011). Health effects of particulate air pollution: a review of epidemiological evidence. Inhal Toxicol.

[2] Brook RD, Rajagopalan S, Pope CA, Brook JR, Bhatnagar A, Diez-Roux AV, Holguin F, Hong Y, Luepker RV, Mittleman MA, Peters A, Siscovick D, Smith SC, Whitsel L, Kaufman JD, American Heart Association Council on Epidemiology and Prevention Council on the Kidney in Cardiovascular Disease and Council on Nutrition Physical Activity and Metabolism (2010). Particulate matter Air pollution and cardiovascular disease: an update to the scientific statement from the American heart association. Circulation.

[3] Franchini M, Mannucci PM (2012). Air pollution and cardiovascular disease. Thromb Res.

[4] Hoek G, Krishnan R, Beelen R, Peters A, Ostro B, Brunekreef B, Kaufman J (2013). Long-term air pollution exposure and cardio- respiratory mortality: a review. Environ Health.

[5] Atkinson RW, Carey IM, Kent AJ, van Staa TP, Anderson HR, Cook DG (2013). Long-term exposure to outdoor air pollution and incidence of cardiovascular diseases. Epidemiology.

[6] Shah AS, Langrish JP, Nair H, McAllister DA, Hunter AL, Donaldson K, Newby DE, Mills NL (2013). Global association of air pollution and heart failure: a systematic review and meta-analysis. Lancet.

[7] Heal MR, Kumar P, Harrison RM (2012). Particles, air quality, policy and health. Chem Soc Rev.

[8] Morawska L, Afshari A, Bae GN, Buonanno G, Chao CYH, Hanninen O, Hofmann W, Isaxon C, Jayaratne ER, Pasanen P, Salthammer T, Waring M, Wierzbicka A (2013). Indoor aerosols: from personal exposure to risk assessment. Indoor Air.

[9] Franck U, Odeh S, Wiedensohler A, Wehner B, Herbarth O (2011). The effect of particle size on cardiovascular disorders-the smaller the worse. Sci Total Environ.

[10] Chen C, Zhao B (2011). Review of relationship between indoor and outdoor particles: I/O ratio, infiltration factor and penetration factor. Atmos Environ.

[11] Rim D, Wallace L, Persily A (2010). Infiltration of outdoor ultrafine particles into a test house. Environ Sci Technol.

[12] Stephens B, Siegel JA (2012). Penetration of ambient submicron particles into single-family residences and associations with building characteristics. Indoor Air.

[13] Bekö G, Weschler CJ, Wierzbicka A, Karottki DG, Toftum J, Loft S, Clausen G (2013). Ultrafine particles: exposure and source apportionment in 56 danish homes. Environ Sci Technol.

[14] Wallace L (2006). Indoor sources of ultrafine and accumulation mode particles: size distributions, size-resolved concentrations, and source strengths. Aerosol Sci Technol.

[15] Hussein T, Glytsos T, Ondracek J, Dohanyosova P, Zdimal V, Hameri K, Lazaridis M, Smolik J, Kulmala M (2006). Particle size characterization and emission rates during indoor activities in a house. Atmos Environ.

[16] Isaxon C, Gudmundson A, Nordin EZ, Lönnblad L, Dahl A, Wieslander G, Bohgard M, Wierzbicka A (2014). Contribution of indooor-generated particles to residential exposure. Atmos Environ.

[17] Mohammadyan M (2012). Personal exposure and indoor home particulate matter: a review. Iranica J Energy Environ.

[18] Bhangar S, Mullen NA, Hering SV, Kreisberg NM, Nazaroff WW (2011). Ultrafine particle concentrations and exposures in seven residences in northern California. Indoor Air.

[19] Wallace L, Ott W (2011). Personal exposure to ultrafine particles. J Expo Sci Environ Epidemiol.

[20] Frampton MW (2006). Inflammation and airborne particles. Clin Occup Environ Med.

[21] Moller P, Mikkelsen L, Vesterdal LK, Folkmann JK, Forchhammer L, Roursgaard M, Danielsen PH, Loft S (2011). Hazard identification of particulate matter on vasomotor dysfunction and progression of atherosclerosis. Crit Rev Toxicol.

[22] Langrish JP, Bosson J, Unosson J, Muala A, Newby DE, Mills NL, Blomberg A, Sandstrom T (2012). Cardiovascular effects of particulate air pollution exposure: time course and underlying mechanisms. J Intern Med.

[23] Weichenthal S (2012). Selected physiological effects of ultrafine particles in acute cardiovascular morbidity. Environ Res.

[24] Buonanno G, Marks GB, Morawska L (2013). Health effects of daily airborne particle dose in children: direct association between personal dose and respiratory health effects. Environ Pollut.

[25] Mills NL, Tornqvist H, Gonzalez MC, Vink E, Robinson SD, Soderberg S, Boon NA, Donaldson K, Sandstrom T, Blomberg A, Newby DE (2007). Ischemic and thrombotic effects of dilute diesel-exhaust inhalation in men with coronary heart disease. N Engl J Med.

[26] Lucking AJ, Lundback M, Barath SL, Mills NL, Sidhu MK, Langrish JP, Boon NA, Pourazar J, Badimon JJ, Gerlofs-Nijland ME, Cassee FR, Boman C, Donaldson K, Sandstrom T, Newby DE, Blomberg A (2011). Particle traps prevent adverse vascular and prothrombotic effects of diesel engine exhaust inhalation in men. Circulation.

[27] Rubinshtein R, Kuvin JT, Soffler M, Lennon RJ, Lavi S, Nelson RE, Pumper GM, Lerman LO, Lerman A (2010). Assessment of endothelial function by non-invasive peripheral arterial tonometry predicts late cardiovascular adverse events. Eur Heart J.

[28] Karottki D, Spilak M, Frederiksen M, Gunnarsen L, Brauner E, Kolarik B, Andersen Z, Sigsgaard T, Barregard L, Strandberg B, Sallsten G, Moller P, Loft S (2013). An indoor air filtration study in homes of elderly: cardiovascular and respiratory effects of exposure to particulate matter. Environ Health.

[29] Brauner EV, Forchhammer L, Moller P, Barregard L, Gunnarsen L, Afshari A, Wahlin P, Glasius M, Dragsted LO, Basu S, Raaschou-Nielsen O, Loft S (2008). Indoor particles affect vascular function in the aged: an air filtration-based intervention study. Am J Respir Crit Care Med.

[30] Allen RW, Carlsten C, Karlen B, Leckie S, Van ES, Vedal S, Wong I, Brauer M (2011). An air filter intervention study of endothelial function among healthy adults in a woodsmoke-impacted community. Am J Respir Crit Care Med.

[31] Ljungman PL, Wilker EH, Rice MB, Schwartz J, Gold DR, Koutrakis P, Vita JA, Mitchell GF, Vasan RS, Benjamin EJ, Mittleman MA, Hamburg NM (2014). Short-term exposure to air pollution and digital vascular function. Am J Epidemiol.

[32] Li Y, Rittenhouse-Olson K, Scheider WL, Mu L (2012). Effect of particulate matter air pollution on C-reactive protein: a review of epidemiologic studies. Rev Environ Health.

[33] Dutta A, Ray MR, Banerjee A (2012). Systemic inflammatory changes and increased oxidative stress in rural Indian women cooking with biomass fuels. Toxicol Appl Pharmacol.

[34] Brauner EV, Moller P, Barregard L, Dragsted LO, Glasius M, Wahlin P, Vinzents P, Raaschou-Nielsen O, Loft S (2008). Exposure to ambient concentrations of particulate air pollution does not influence vascular function or inflammatory pathways in young healthy individuals. Part Fibre Toxicol.

[35] Buonanno G, Stabile L, Morawska L (2014). Personal exposure to ultrafine particles: the influence of time-activity patterns. Sci Total Environ.

[36] Vinzents PS, Moller P, Sorensen M, Knudsen LE, Hertel O, Jensen FP, Schibye B, Loft S (2005). Personal exposure to ultrafine particles and oxidative DNA damage. Environ Health Perspect.

[37] Gonzalez-Quintela A, Dam Laursen AS, Vidal C, Skaaby T, Gude F, Linneberg A (2014). IgE antibodies to alpha-gal in the general adult population. Relationship with tick bites, atopy, and cat ownership. Clin Exp Allergy.

[38] Miller MR, Hankinson J, Brusasco V, Burgos F, Casaburi R, Coates A, Crapo R, Enright P, van der Grinten CP, Gustafsson P, Jensen R, Johnson DC, Macintyre N, McKay R, Navajas D, Pedersen OF, Pellegrino R, Viegi G, Wanger J (2005). Standardisation of spirometry. Eur Respir J.

[39] Weichenthal S, Mallach G, Kulka R, Black A, Wheeler A, You H, St-Jean M, Kwiatkowski R, Sharp D (2013). A randomized double-blind crossover study of indoor air filtration and acute changes in cardiorespiratory health in a First Nations community. Indoor Air.

[40] Pope CA, Hansen JC, Kuprov R, Sanders MD, Anderson MN, Eatough DJ (2011). Vascular function and short-term exposure to fine particulate air pollution. J Air Waste Manag Assoc.

[41] Forchhammer L, Moller P, Riddervold IS, Bonlokke J, Massling A, Sigsgaard T, Loft S (2012). Controlled human wood smoke exposure: oxidative stress, inflammation and microvascular function. Part Fibre Toxicol.

[42] Karottki DG, Beko G, Clausen G, Madsen AM, Andersen ZJ, Massling A, Ketzel M, Ellermann T, Lund R, Sigsgaard T, Moller P, Loft S (2014). Cardiovascular and lung function in relation to outdoor and indoor exposure to fine and ultrafine particulate matter in middle-aged subjects. Environ Int.

[43] McCreanor J, Cullinan P, Nieuwenhuijsen MJ, Stewart-Evans J, Malliarou E, Jarup L, Harrington R, Svartengren M, Han IK, Ohman-Strickland P, Chung KF, Zhang J (2007). Respiratory effects of exposure to diesel traffic in persons with asthma. N Engl J Med.

[44] Ruckerl R, Phipps R, Schneider A, Frampton M, Cyrys J, Oberdorster G, Wichmann HE, Peters A (2007). Ultrafine particles and platelet activation in patients with coronary heart disease - results from a prospective panel study. Part Fibre Toxicol.

[45] Bruske I, Hampel R, Socher MM, Ruckerl R, Schneider A, Heinrich J, Oberdorster G, Wichmann HE, Peters A (2010). Impact of ambient air pollution on the differential white blood cell count in patients with chronic pulmonary disease. Inhal Toxicol.

[46] Pope CA, Hansen ML, Long RW, Nielsen KR, Eatough NL, Wilson WE, Eatough DJ (2004). Ambient particulate air pollution, heart rate variability, and blood markers of inflammation in a panel of elderly subjects. Environ Health Perspect.

[47] Zuurbier M, Hoek G, Oldenwening M, Meliefste K, Krop E, van den Hazel P, Brunekreef B (2011). In-traffic air pollution exposure and CC16, blood coagulation, and inflammation markers in healthy adults. Environ Health Perspect.

[48] Gong H, Linn WS, Clark KW, Anderson KR, Sioutas C, Alexis NE, Cascio WE, Devlin RB (2008). Exposures of healthy and asthmatic volunteers to concentrated ambient ultrafine particles in Los Angeles. Inhal Toxicol.

[49] Soyer OU, Akdis M, Ring J, Behrendt H, Crameri R, Lauener R, Akdis CA (2013). Mechanisms of peripheral tolerance to allergens. Allergy.

[50] Linneberg A, Nielsen NH, Madsen F, Frolund L, Dirksen A, Jorgensen T (2001). Smoking and the development of allergic sensitization to aeroallergens in adults: a prospective population-based study. The Copenhagen Allergy Study. Allergy.

[51] Folino AF, Scapellato ML, Canova C, Maestrelli P, Bertorelli G, Simonato L, Iliceto S, Lotti M (2009). Individual exposure to particulate matter and the short-term arrhythmic and autonomic profiles in patients with myocardial infarction. Eur Heart J.

[52] Sorensen M, Schins RP, Hertel O, Loft S (2005). Transition metals in personal samples of PM2.5 and oxidative stress in human volunteers. Cancer Epidemiol Biomarkers Prev.

[53] Weichenthal SA, Godri-Pollitt K, Villeneuve PJ (2013). PM2.5, oxidant defence and cardiorespiratory health: a review. Environ Health.

[54] Sacks JD, Stanek LW, Luben TJ, Johns DO, Buckley BJ, Brown JS, Ross M (2011). Particulate matter-induced health effects: who is susceptible?. Environ Health Perspect.

